# Velocity gradient dependent structures of 12–2–12 wormlike micelles: insights from small-angle neutron scattering in the 1–2 shear plane

**DOI:** 10.1107/S1600576725006521

**Published:** 2025-07-29

**Authors:** Hiroki Iwase, Shin-ichi Takata, Toshiaki Morikawa, Tomokazu Yoshimura

**Affiliations:** a Neutron Science and Technology Center Comprehensive Research Organization for Science and Society (CROSS) 162-1 Shirakata Tokai Ibaraki319-1106 Japan; b Materials and Life Science Division, J-PARC Center Japan Atomic Energy Agency 2-4 Shirakata Tokai Ibaraki319-1195 Japan; c Department of Chemistry, Faculty of Science Nara Women’s University Kitauoyanishi-machi Nara630-8506 Japan

**Keywords:** small-angle neutron scattering, SANS, rheology, velocity–velocity gradient

## Abstract

The velocity gradient dependent shear-induced structural evolution of wormlike micelles (WLMs) formed by a gemini-type cationic surfactant (12–2–12) in an aqueous solution was investigated using small-angle neutron scattering (SANS). To achieve this, a cell for observation in the velocity–velocity gradient (1–2) shear plane was constructed. SANS measurements were conducted at multiple positions across the gap of this 1–2 shear cell under various shear rates, covering both shear-thickening and shear-thinning regimes. During shear thickening at apparent shear rates of 70–200 s^−1^, a clear position-dependent alignment of WLMs was observed, with higher orientation near the rotor. In contrast, the intermicellar distance remained largely uniform across the gap. Neutron transmission measurements indicated no significant macroscopic concentration fluctuations. These results suggest that shear thickening is primarily driven by shear-induced micellar elongation and orientation, rather than the formation of concentration fluctuations. This study indicates the utility of SANS measurements in the 1–2 shear plane in revealing spatially resolved structural details, providing crucial insights into the 3D behavior of complex fluids under shear and complementing previous Rheo-SANS findings.

## Abstract 2

A new 1–2 plane shear cell has been developed to observe structural changes and concentration fluctuations under flow fields depending on the velocity gradient, which cannot be measured by the Rheo-SANS small-angle neutron scattering technique.

## 1. Introduction

Rheological measurements are fundamental to various industrial applications, including food, cosmetics, pharmaceuticals and polymeric materials, where controlling the material deformation and flow properties is crucial for product quality and performance. Complex fluids, such as colloidal dispersions and polymer solutions, exhibit diverse rheological behaviors under shear flow. A thorough understanding of these behaviors is crucial for optimizing industrial processes and enhancing product quality (Wagner & Mewis, 2021 ▸).

Wormlike micelles (WLMs), formed by surfactants, are notable for their nonlinear rheological behaviors (shear thinning and shear thickening) under shear flow (Dreiss & Feng, 2017 ▸). This distinct rheology is closely linked to hierarchical structural changes spanning the nanoscale to the mesoscale under shear stress, which include micellar elongation, orientation, branching, the formation of higher-order structures and concentration inhomogeneities. Consequently, nanoscale structural analysis under shear flow is crucial for elucidating these phenomena. Small-angle neutron scattering (SANS) is a powerful technique for these investigations. The Rheo-SANS method, which combines rheological measurements with SANS, is particularly effective in revealing the relationship between macroscopic physical properties and nanostructures.

Numerous Rheo-SANS studies on WLMs formed by cationic surfactants [*e.g.* cetylpyridinium chloride (CPyCl) (Mütze *et al.*, 2014 ▸; Gurnon, Lopez-Barron *et al.*, 2014 ▸; Herle *et al.*, 2007 ▸), cetyltri­methyl­ammonium bromide (Lutz-Bueno *et al.*, 2013 ▸; Takeda *et al.*, 2011 ▸; Dehmoune *et al.*, 2009 ▸), cetyltri­methyl­ammonium tosyl­ate (Truong & Walker, 2002 ▸; Berret *et al.*, 2001 ▸; Truong & Walker, 2000 ▸; Berret *et al.*, 1998 ▸)] with organic salts have demonstrated shear-induced orientations and phase transitions (Helgeson *et al.*, 2010 ▸). Additionally, CPyCl/sodium salicylate systems have been shown to have shear-induced structures (Helgeson *et al.*, 2009 ▸). Previously, we constructed a Rheo-SANS system (Iwase *et al.*, 2019 ▸) to study the interplay between rheological behavior and nano­structures in fluid materials, such as surfactant solutions (Nemoto *et al.*, 2024 ▸; Uyama *et al.*, 2022 ▸; Iwase, Kawai *et al.*, 2021 ▸; Sugahara *et al.*, 2020 ▸), polymer solutions (Gupit *et al.*, 2020 ▸), inorganic suspensions (Kajiyama *et al.*, 2020 ▸) and clay suspensions (Iwase, Kubota *et al.*, 2021 ▸; Iwase *et al.*, 2018 ▸).

**Figure 1:**
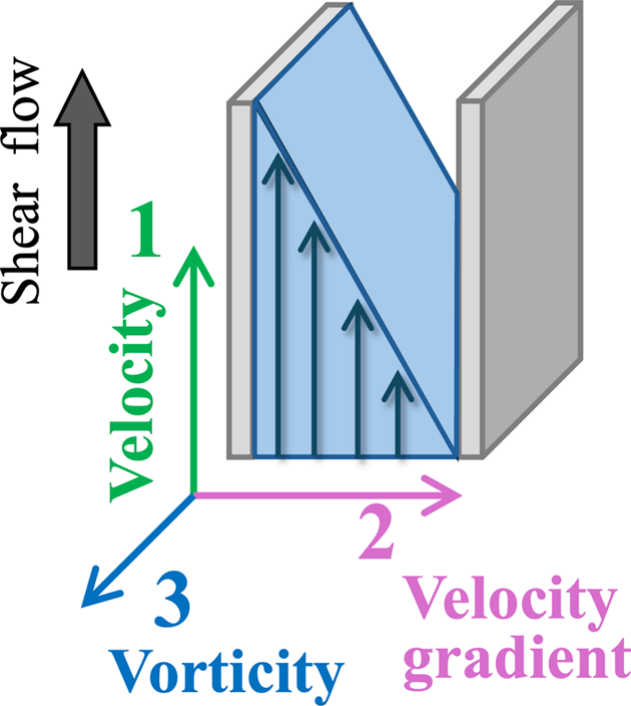
Schematic diagram of a coordinate system defined by (1) velocity, (2) velocity gradient and (3) vorticity for SANS.

To understand the rheological behavior of materials under shear flow, structural characterization in three different shear planes – velocity, velocity gradient and vorticity – is required (Fig. 1 ▸). Rheo-SANS enables us to observe structures in the velocity–vorticity (1–3) and velocity gradient–vorticity (2–3) shear planes. However, structures in the velocity–velocity gradient (1–2) shear plane have remained inaccessible, limiting our ability to observe the structural changes influenced by the velocity gradients.

It has recently been reported that particle orientation and concentration fluctuations depend on the velocity gradient and are involved in peculiar rheological behavior. In particular, macroscopic concentration fluctuations, referred to as shear bands, have been observed in various systems and are a key area of interest (Huang *et al.*, 2025 ▸; Bassu *et al.*, 2024 ▸; Korculanin *et al.*, 2021 ▸; Lang, Kohlbrecher *et al.*, 2019 ▸; Calabrese *et al.*, 2016 ▸; Sambasivam *et al.*, 2015 ▸; Min Kim *et al.*, 2014 ▸; López-Barrón *et al.*, 2014 ▸; Fielding, 2014 ▸; Kunita *et al.*, 2012 ▸; Helgeson *et al.*, 2010 ▸; Furukawa & Tanaka, 2009 ▸; Miller & Rothstein, 2007 ▸; Hu & Lips, 2005 ▸; Lettinga & Dhont, 2004 ▸; Salmon *et al.*, 2003 ▸; Decruppe *et al.*, 2001 ▸; Liu & Pine, 1996 ▸). Consequently, experimental setups capable of observing structures in the 1–2 shear plane are essential for a complete understanding of material behavior under shear flow (Lang, Porcar *et al.*, 2019 ▸; Eberle & Porcar, 2012 ▸). While Rheo-SANS systems have been developed in multiple neutron scattering facilities, cells specifically designed to observe structure in the 1–2 shear plane remain relatively rare (Velichko *et al.*, 2019 ▸; Gurnon, Godfrin *et al.*, 2014 ▸; Eberle & Porcar, 2012 ▸; Liberatore *et al.*, 2006 ▸; Van der Werff *et al.*, 1990 ▸).

In this study, a 1–2 shear cell was utilized to investigate the shear-induced structural behavior of the gemini-type cationic surfactant 12–2–12, with a particular focus on elucidating spatially resolved structural behaviors in the 1–2 shear plane. The aim of this study is to provide new insights into the 3D response to shear flow by complementing our previous Rheo-SANS investigations (Iwase *et al.*, 2019 ▸), which characterized structures in the 1–3 and 2–3 shear planes. The 12–2–12 system, which has been observed to form WLMs and exhibit both shear-thinning and shear-thickening behaviors, serves as an ideal model to explore the structural origins of these complex rheological phenomena through spatially resolved SANS measurements in the 1–2 shear plane.

## 2. Materials and methods

### 2.1. Samples

**Figure 2:**
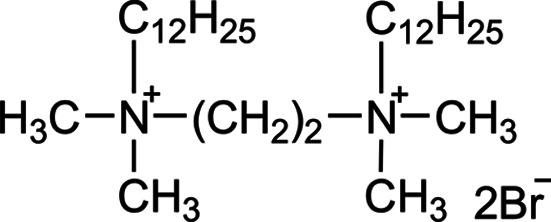
Chemical structure of the gemini-type cationic surfactant (12–2–12).

The gemini surfactant 12–2–12 consists of two cationic amphiphilic units [C_12_H_25_N^+^(CH_3_)_2_Br^−^] connected by a spacer, as shown in Fig. 2 ▸. The compound was synthesized following a previously reported procedure (Esumi *et al.*, 1996 ▸; Zana & Talmon, 1993 ▸). A 12–2–12 solution was prepared in D_2_O with a volume fraction (φ) of 0.0135. In salt-free solutions, 12–2–12 undergoes micellar transitions, forming spherical micelles at low concentrations and transitioning to rod-like and wormlike micelles as concentration increases (In *et al.*, 2010 ▸; Oda *et al.*, 1997 ▸). Further increases in concentration extend micellar lengths. Previous Rheo-SANS studies have demonstrated shear-thinning and -thickening behavior in this system at 30°C (Iwase *et al.*, 2019 ▸).

### 2.2. Rheological measurements

Rheological measurements were conducted using an MCR302 stress-controlled rheometer (Anton Paar, Graz, Austria). A cone-plate geometry (*e.g.* 50 mm diameter, 1° angle, 0.100 mm truncation) was utilized. The shear rate was varied from 10 to 900 s^−^^1^, while the sample temperature was maintained at 30°C using a Peltier temperature controller.

### 2.3. SANS

SANS measurements were conducted using the small- and wide-angle neutron scattering instrument (TAIKAN) installed at the Materials and Life Science Experimental Facility (MLF) of the Japan Proton Accelerator Research Complex (J-PARC), Tokai, Japan (Takata *et al.*, 2015 ▸). TAIKAN has four detector banks (small-angle, middle-angle, high-angle and backward). The small-angle detector bank, located 5.65 m from the sample position, had a large effective area of ∼2.2 × 2.3 mm, excluding a 64 × 64 mm central area. A low-efficiency N_2_ monitor positioned behind the center of the small-angle detector bank measured transmission simultaneously with scattering. Using white neutron beams with a wavelength (λ) range of 1.0–7.5 Å based on the time-of-flight (TOF) method, a *Q* range from 5 × 10^−3^ to 17 Å^−1^ could be covered in a single measurement [the magnitude of the scattering vector *Q* is defined as *Q* = 4π sin(θ/2)/λ, with θ the scattering angle]. However, in this study, the observed *Q* range was constrained to 0.01 < *Q* < 1 Å^−1^ due to instrumental background at lower *Q* and low accuracy of the scattering intensity from the sample at higher *Q*.

**Figure 3:**
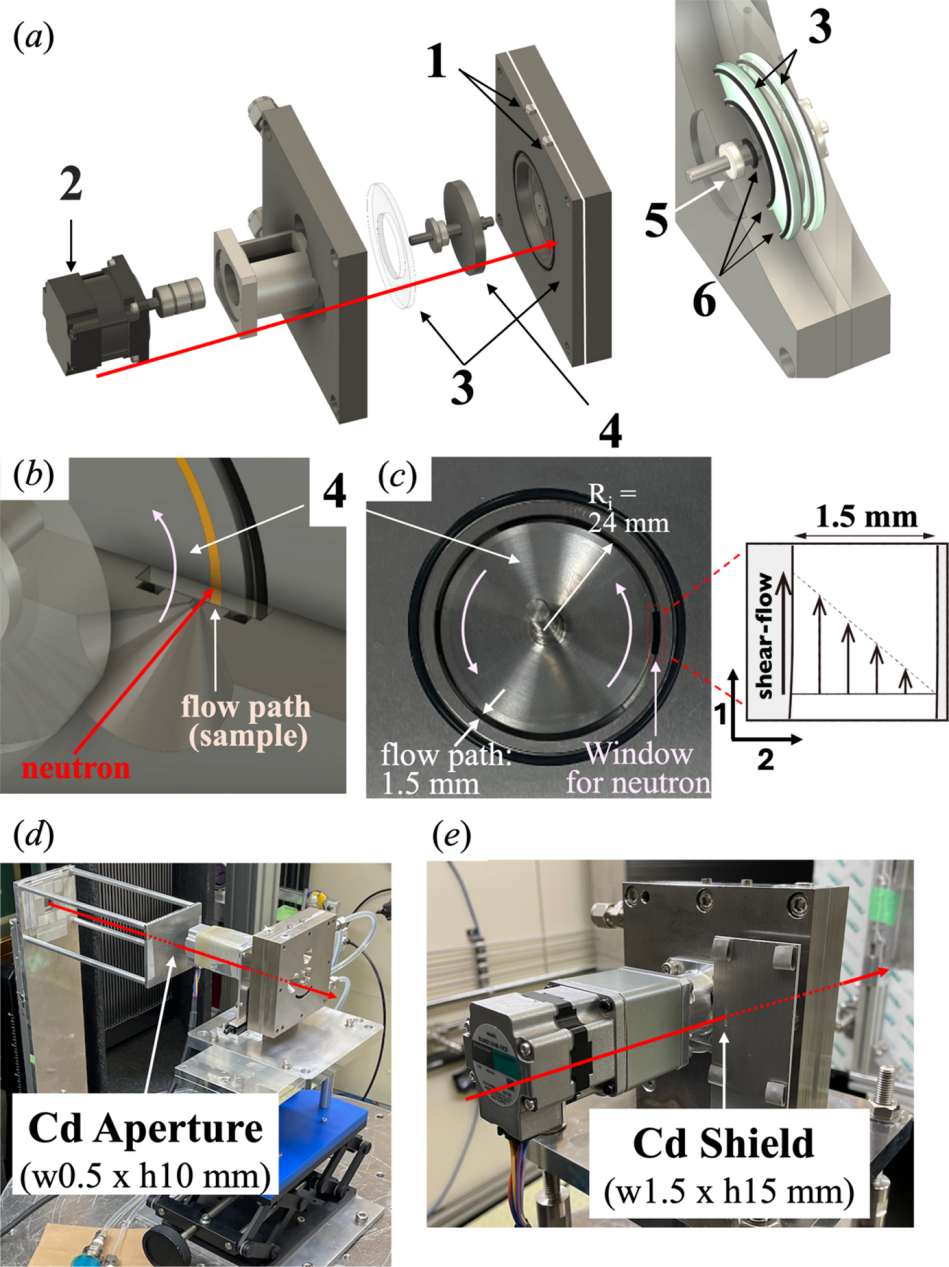
(*a*), (*b*) Schematic drawing of the 1–2 shear cell. (1) Sample inlets, (2) a brushless motor, (3) donut-shaped quartz glass windows (thickness of 3 mm), (4) a rotor, (5) a bearing, (6) O-rings. Photographs of (*c*) the rotor and neutron-irradiated area (dashed red window), (*d*) the Cd aperture, and (*e*) the Cd shield.

The 12–2–12 solution was investigated under shear flow using a recently constructed 1–2 shear cell. As illustrated in Fig. 3 ▸, the 1–2 shear cell allows for structural observation in the velocity–velocity gradient (1–2) shear plane. The cell was fabricated from a Ti–6 Al–4 V (Ti-64) titanium alloy, a material selected for its exceptional corrosion resistance. The system design was informed by prior successful implementations in the field (Gurnon, Godfrin *et al.*, 2014 ▸; Liberatore *et al.*, 2006 ▸), notably incorporating a liquid seal concept. The rotor, with an inner diameter of 50.0 mm, featured a flow channel width (gap) of 1.5 mm and a path length (sample thickness) of 6 mm. Shear flow was generated using a brushless motor (BLM015HK-5CS, Oriental Motor Co. Ltd) operated with a 1:5 gear ratio. Brushless motors are known for their high operational speeds and low levels of vibration. These motors are characterized by a specified rotation speed range of 100–3000 r min^−1^, enabling access to a shear rate (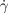) range of 40–1000 s^−1^ for the current experiments. The apparent shear rate was related to the motor angular velocity (Ω) via equation (1), based on assumptions consistent with double-cylindrical Couette cell configurations (JIS Z 8803, 1991 ▸):
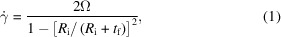
where *R*_i_ (mm) is the rotor radius and *t*_f_ (mm) is the flow channel width. Note that the shear cell is designed with the potential to achieve higher shear rates, up to approximately 5000 s^−1^, through the implementation of an alternative motor or gear configuration. However, such capabilities were not explored in the present study. The 1–2 shear cell cannot directly measure shear stress because it lacks torque sensors. Therefore, Rheo-SANS measurements are necessary to investigate the relationship between rheological behavior and nanostructure. The study employed martensitic stainless-steel bearings (TBN-6H, NSK Micro Precision Co. Ltd, Tokyo, Japan). Liquid seals, fabricated from Viton O-rings, were strategically positioned between the bearings and the donut-shaped quartz glass windows (3 mm thickness). The sample was inserted into the cell by means of a tubing pump (Peristaltic Pump, Atto Corporation, Tokyo, Japan).

A sample aperture (w0.5 × h10 mm) was positioned on an *X*–*Z* movable bench 150 mm upstream of the sample [Fig. 3 ▸(*d*)]. The *X*–*Z* movable bench is usually used to adjust the standard sample aperture. Additionally, a manually operated Cd shield (w1.5 × h15 mm) was placed directly in front of the sample window [Fig. 3 ▸(*e*)]. Parasitic scattering is influenced by the sample aperture size, first aperture size and collimation length (CL), accounting for beam divergence. For measurements on the TAIKAN instrument, the first aperture size was set to 8 × 23 mm, with a CL of 7.55 m (for a 1–2 shear SANS measurement). The aperture size was similar to that previously used in Rheo-SANS measurement (tangential position). Due to the significantly reduced sample width required for these 1–2 plane measurements, a proven optical system designed for a 0.5 mm beam width was initially employed to ensure reliable performance.

The shear cell operation and data acquisition on the TAIKAN instrument were managed using the instrument software framework *IROHA2* developed by J-PARC MLF (Hasemi *et al.*, 2024 ▸). This framework supports automated measurements and system status monitoring, ensuring reliable and reproducible data collection. The apparent shear rates were incrementally increased from 0 to 900 s^−1^, with simultaneous SANS and transmission measurements at each step. The exposure time for each measurement was 15 min, and the sample temperature was maintained at 30°C using temperature-controlled circulating water.

TOF data were corrected for wavelength dependence using measurements from an empty cell. The scattering intensity *I*(*Q*) was obtained by sector averaging data along the vertical and horizontal directions with an azimuthal angle of ±15°. Parasitic scattering from the cell wall was removed analytically by pixel masking. Data reduction and analysis were performed using the *UTSUSEMI* software package (Inamura *et al.*, 2013 ▸). Absolute scattering intensity calibration was performed in the following manner. First, SANS measurements of a glassy carbon secondary standard (Zhang *et al.*, 2010 ▸) and the 12–2–12 solution inserted into a quartz cell were conducted using standard pinhole collimation on TAIKAN (10 mm-diameter sample aperture) to obtain their absolute scattering intensities per unit sample volume. Subsequently, SANS measurements of the 12–2–12 solution in a stationary state were performed at each of the four positions within the 1–2 shear cell using the 0.5 mm-wide neutron beam. The *Q* dependence of these SANS profiles was found to be identical to that obtained with the standard pinhole collimation. Therefore, by comparing these datasets, the data from the 1–2 shear cell measurements were scaled to absolute units. This procedure also allowed for an estimation of the effective irradiated area at each measurement position within the 1–2 shear cell.

## 3. Results and discussion

### 3.1. Shear rate dependence

#### 3.1.1. Rheological measurements

**Figure 4:**
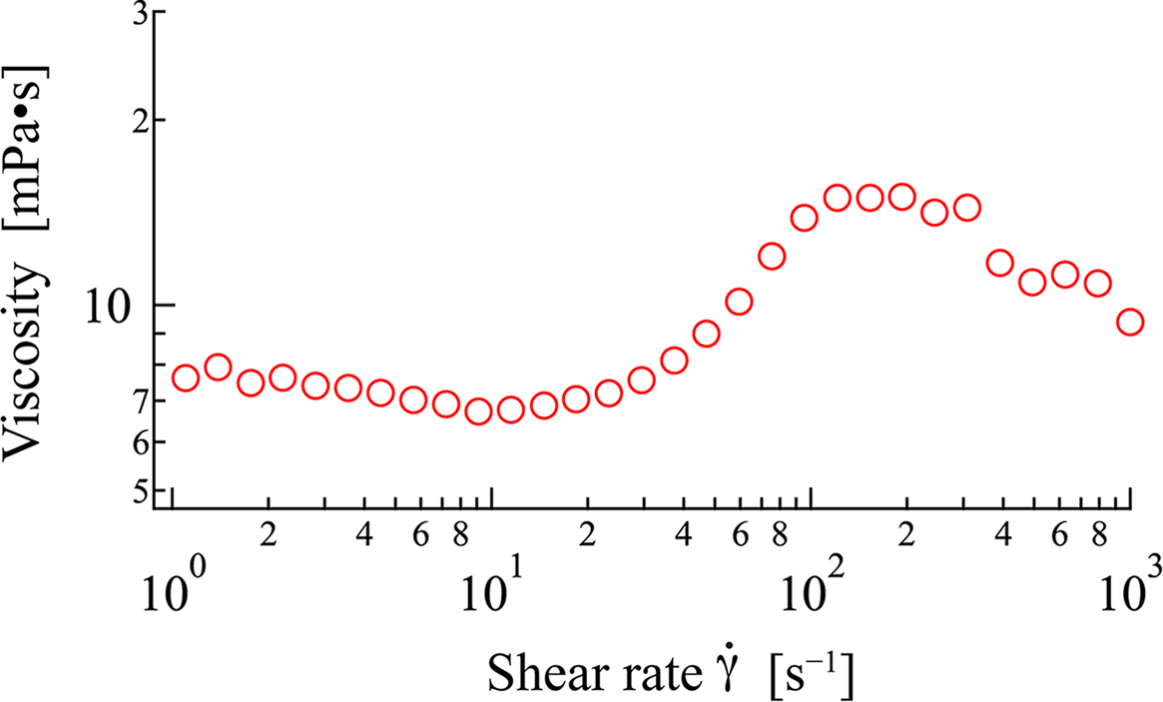
Shear rate dependence of the viscosity of 12–2–12 in an aqueous solution (D_2_O) at 30°C. The volume fraction (φ) is 0.0135.

Fig. 4 ▸ illustrates the 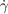 dependence of the viscosity of 12–2–12 in an aqueous solution at φ = 0.0135 at 30°C, as measured using a cone-plate geometry. At 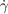 < 40 s^−1^, the apparent viscosity was relatively low. The viscosity increased within the range of 40–150 s^−1^, showing shear-thickening behavior. At higher shear rates (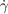 > 300 s^−1^), a decrease in viscosity, known as shear thinning, was observed. These rheological behaviors observed for 12–2–12 in an aqueous solution with the cone-plate geometry are qualitatively consistent with previously reported results, including our previous Rheo-SANS results (Iwase *et al.*, 2019 ▸). While the general trend is similar, quantitative comparisons of critical shear rates or the magnitude of viscosity changes should consider the differences in measurement geometries (cone-plate here versus Couette or parallel-plate in other studies or in SANS cells). For instance, slight variations in the onset of shear thickening or the rate of viscosity increase compared with measurements in different geometries might be expected. Consequently, these rheological characteristics, determined under well defined shear conditions, provide the necessary context for interpreting the SANS results obtained under shear.

#### 3.1.2. SANS

**Figure 5:**
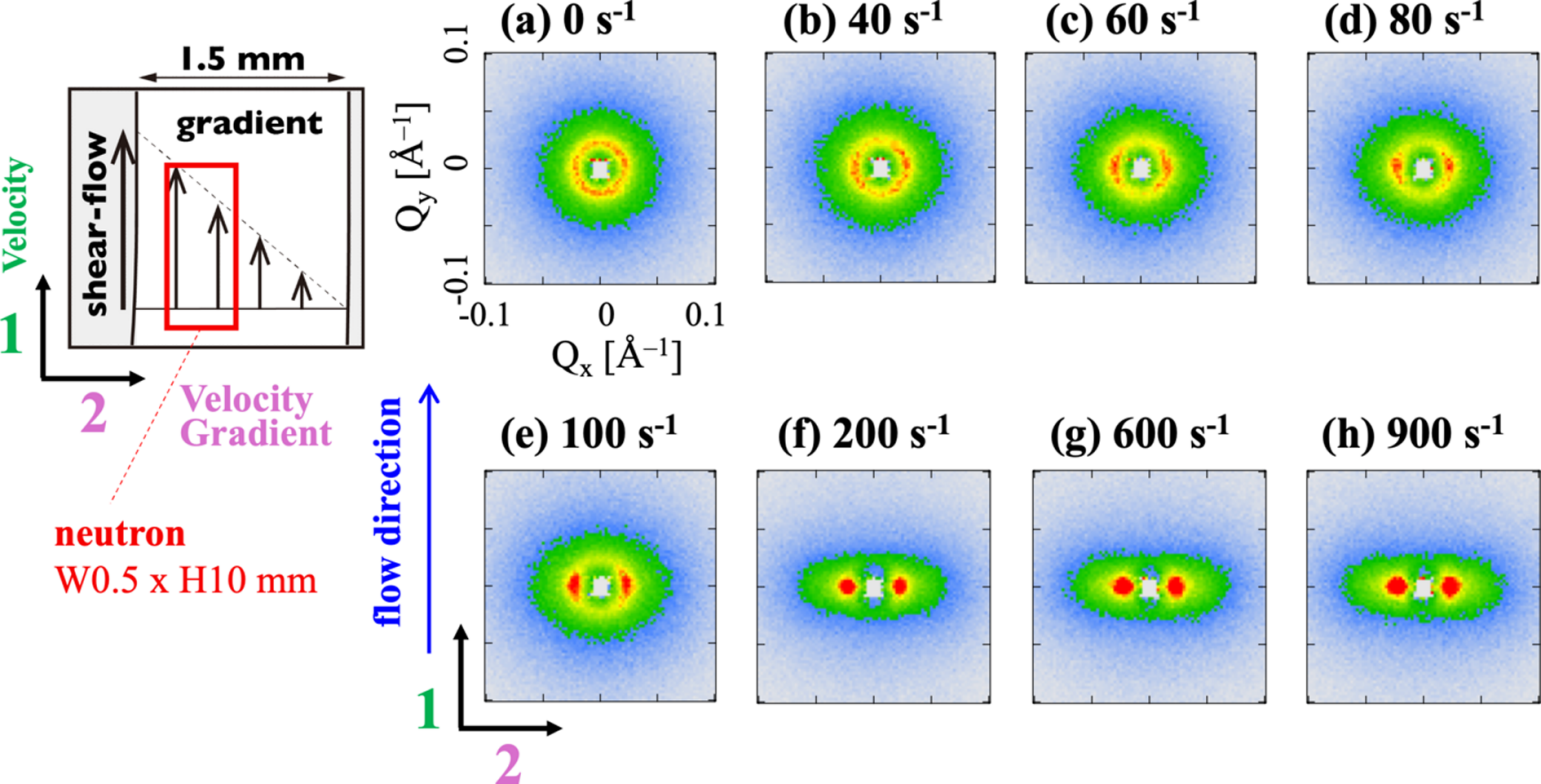
2D SANS profiles *I*(*Q*_*x*_, *Q*_*y*_) for 12–2–12 in an aqueous solution at a volume fraction (φ) of 0.0135. (*a*) 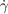 = 0 s^−1^, (*b*) 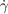 = 40 s^−1^, (*c*) 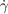 = 60 s^−1^, (*d*) 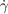 = 80 s^−1^, (*e*) 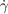 = 100 s^−1^, (*f*) 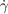 = 200 s^−1^, (*g*) 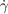 = 600 s^−1^, (*h*) 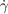 = 900 s^−1^.

Fig. 5 ▸ shows 2D SANS profiles for 12–2–12 in an aqueous solution at φ = 0.0135 under varying shear rates ranging from 0 to 900 s^−1^. The 2D SANS profiles were approximately isotropic at lower shear rates (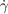 < 40 s^−1^), indicating a random orientation of WLMs. In contrast, at relatively high shear rates (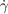 > 100 s^−1^), the 2D SANS profiles were anisotropic, with strong scattering observed perpendicular to the shear flow. This anisotropy is attributed to the orientation of WLMs in the flow direction, consistent with previous Rheo-SANS studies (Iwase *et al.*, 2019 ▸).

**Figure 6:**
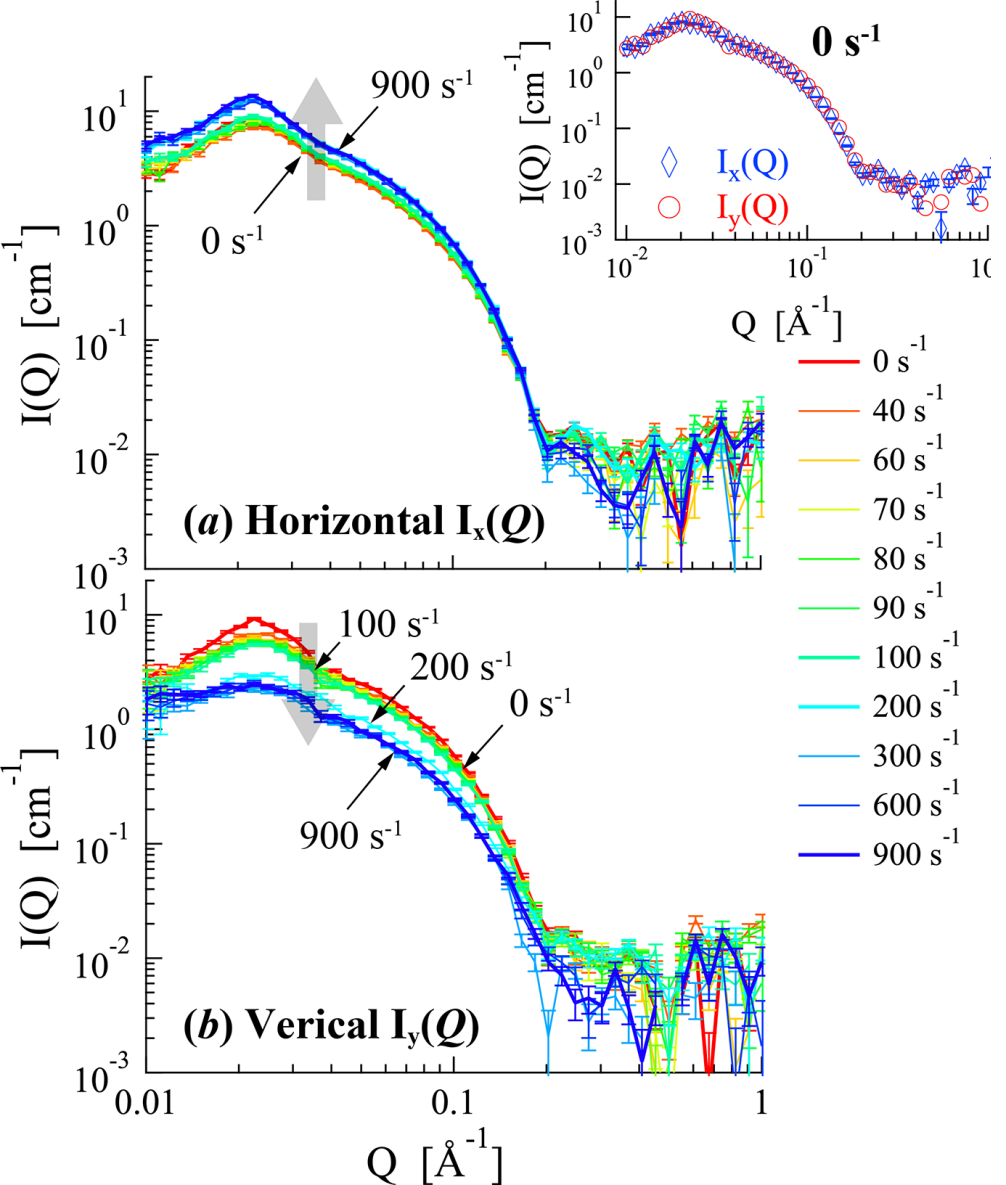
Shear rate dependence of the sector-averaged SANS profiles for 12–2–12 in an aqueous solution at a volume fraction (φ) of 0.0135. (*a*) Horizontal and (*b*) vertical SANS profiles which were, respectively, perpendicular and parallel to the flow. The 2D SANS profiles (in Fig. 5 ▸) were sector-averaged with the azimuthal angle of ±15°. The inset of Fig. 6 ▸(*a*) presents the sector-average SANS profiles for 12–2–12 in an aqueous solution under stationary states (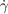 = 0 s^−1^).

To further analyze these structural changes, 1D SANS profiles were obtained by sector averaging the 2D SANS profile. Fig. 6 ▸ shows the sector-averaged SANS profiles for 12–2–12 in an aqueous solution. The horizontal [*I*_*x*_(*Q*)] and vertical [*I*_*y*_(*Q*)] SANS profiles were perpendicular and parallel to the flow, respectively. Under stationary conditions (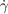 = 0 s^−1^), *I*_*x*_(*Q*) and *I*_*y*_(*Q*) were identical within experimental uncertainty (see inset of Fig. 6 ▸), indicating isotropic scattering. All SANS profiles for 12–2–12 in an aqueous solution exhibited broad peaks in the *Q* range of 0.015–0.04 Å^−1^, arising from electrostatic repulsions between the surface charges of the micelles. The peak position indicates the intermicellar distance. In the horizontal direction, the SANS profiles *I*_*x*_(*Q*) in the *Q* range of 0.01 < *Q* < 0.2 Å^−1^ showed slight changes near 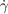
 = 200 s^−1^, with an increase in peak intensity. In contrast, the vertical SANS intensity *I*_*y*_(*Q*) decreased, and its peak profile broadened within the same shear rate range. The observed changes in the horizontal and vertical SANS profiles with increasing shear rates were attributed to the orientation of the WLMs along the flow direction (corresponding to the vertical direction in the profiles). A more quantitative analysis of this orientation is provided by the alignment factor discussed subsequently.

To quantitatively evaluate the degree of orientation of the WLMs, the alignment factor (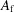) was estimated using the following equation (Angelico *et al.*, 2010 ▸; Borse *et al.*, 2004 ▸; Iwase *et al.*, 2019 ▸):
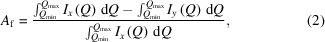
where *I*_*x*_(*Q*) and *I*_*y*_(*Q*) are the SANS intensities perpendicular and parallel to the flow, respectively. The 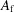 values were evaluated using *Q*_min_ = 0.015 Å^−1^ and *Q*_max_ = 0.06 Å^−1^.

**Figure 7:**
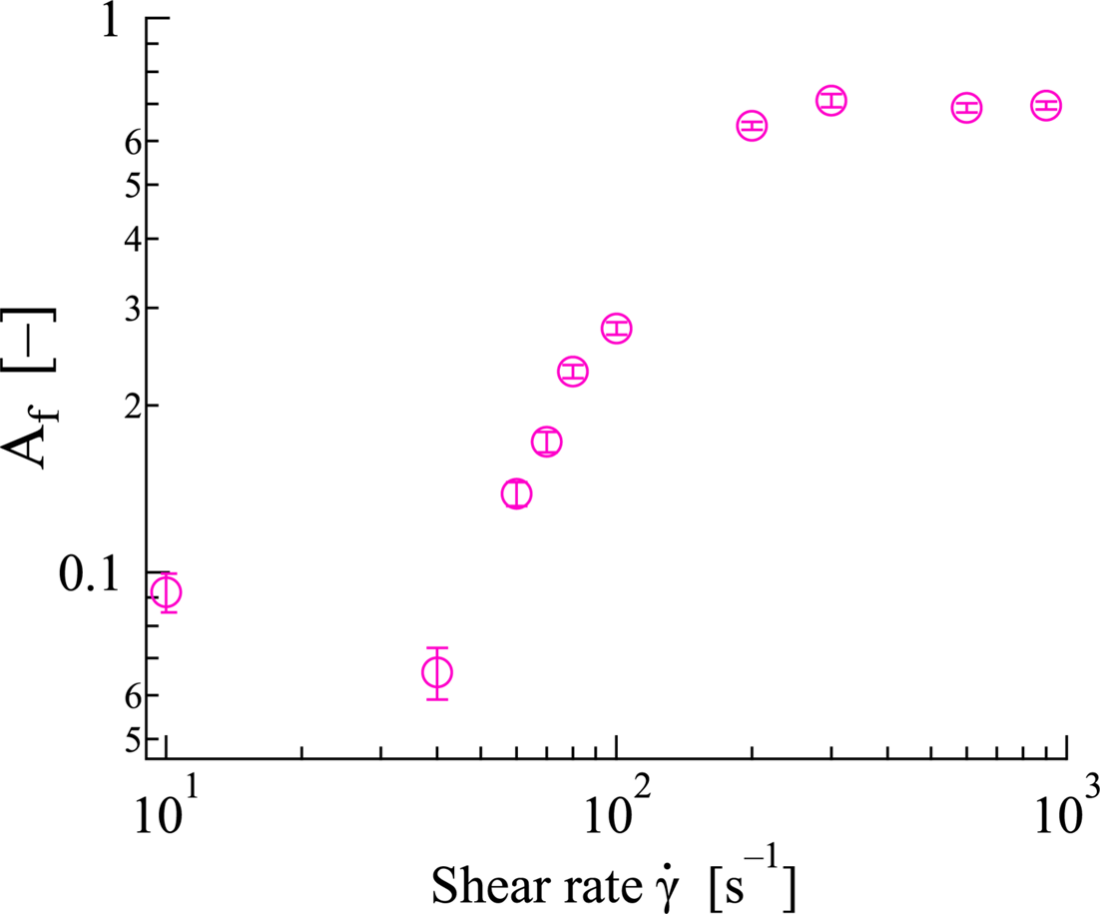
Shear rate dependence of the alignment factor 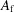. The 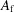 value was obtained by the 1D SANS profiles.

Fig. 7 ▸ shows the shear rate dependence of 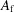. At 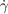 < 60 s^−1^, the 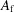 values remained consistently low, indicating that the WLMs were randomly distributed throughout the solution. When the shear rate was increased from 60 to 200 s^−1^, the 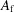 values gradually increased from 0 to ∼0.55. This behavior was observed near the shear thickening, indicating that the micelle orientation and shear thickening occurred in the same shear rate region. Furthermore, in the range of 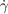 > 200 s^−1^, where shear thinning was observed, the 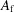 values remained nearly constant. The orientational behaviors of the WLMs formed by the 12–2–12 solution in the 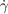 = 70–200 s^−1^ (shear-thickening) and >200 s^−1^ (shear-thinning) regions were consistent with the results obtained in previous Rheo-SANS studies on the same 12–2–12 in an aqueous solution at φ = 0.0135, suggesting that the 1–2 shear cell is capable of applying the intended shear field and detecting the expected structural response.

### 3.2. Position (velocity gradient) dependence

#### 3.2.1. SANS

**Figure 8:**
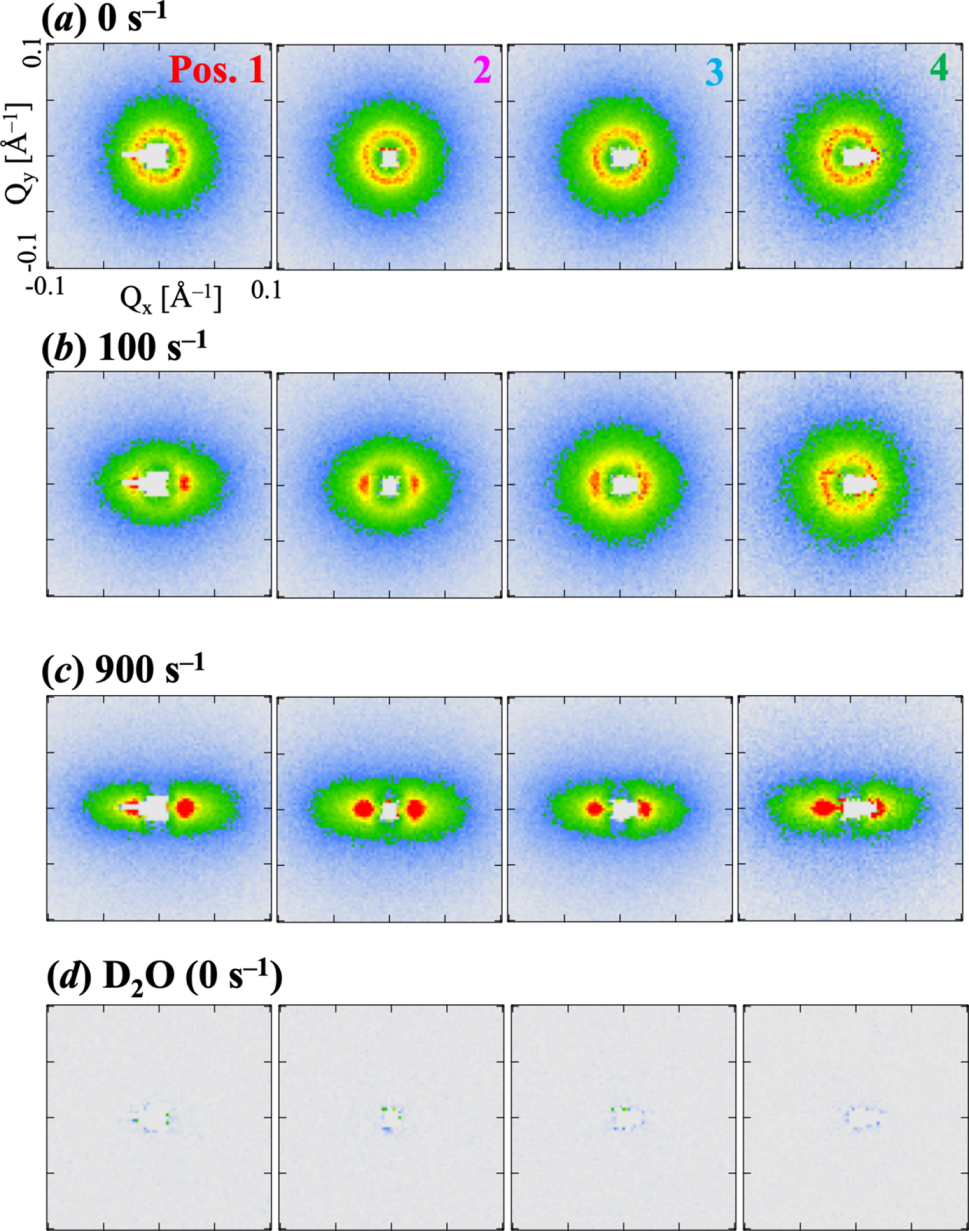
Position dependence of 2D SANS profiles *I*(*Q*_*x*_, *Q*_*y*_) for 12–2–12 in an aqueous solution at a volume fraction (φ) of 0.0135 at (*a*) 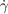 = 0 s^−1^, (*b*) 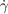 = 100 s^−1^, (*c*) 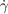 = 900 s^−1^, (*d*) D_2_O (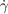 = 0 s^−1^).

To investigate the position-dependent structural behavior of WLMs formed by 12–2–12 under shear flow within the 1–2 shear plane, SANS measurements were performed at four different positions across the 1.5 mm gap of the flow channel. The centers of these positions were separated by approximately 0.23 mm. Fig. 8 ▸ shows the position dependence of the 2D SANS profiles for the 12–2–12 solution at φ = 0.0135 at 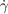 = (*a*) 0, (*b*) 100 and (*c*) 900 s^−1^. The 2D SANS for D_2_O is also shown in Fig. 8 ▸(*d*), indicating that the parasitic scattering from the 1–2 shear cell wall has been removed. Note that the profiles for position 2 are identical to those shown in Fig. 5 ▸.

**Figure 9:**
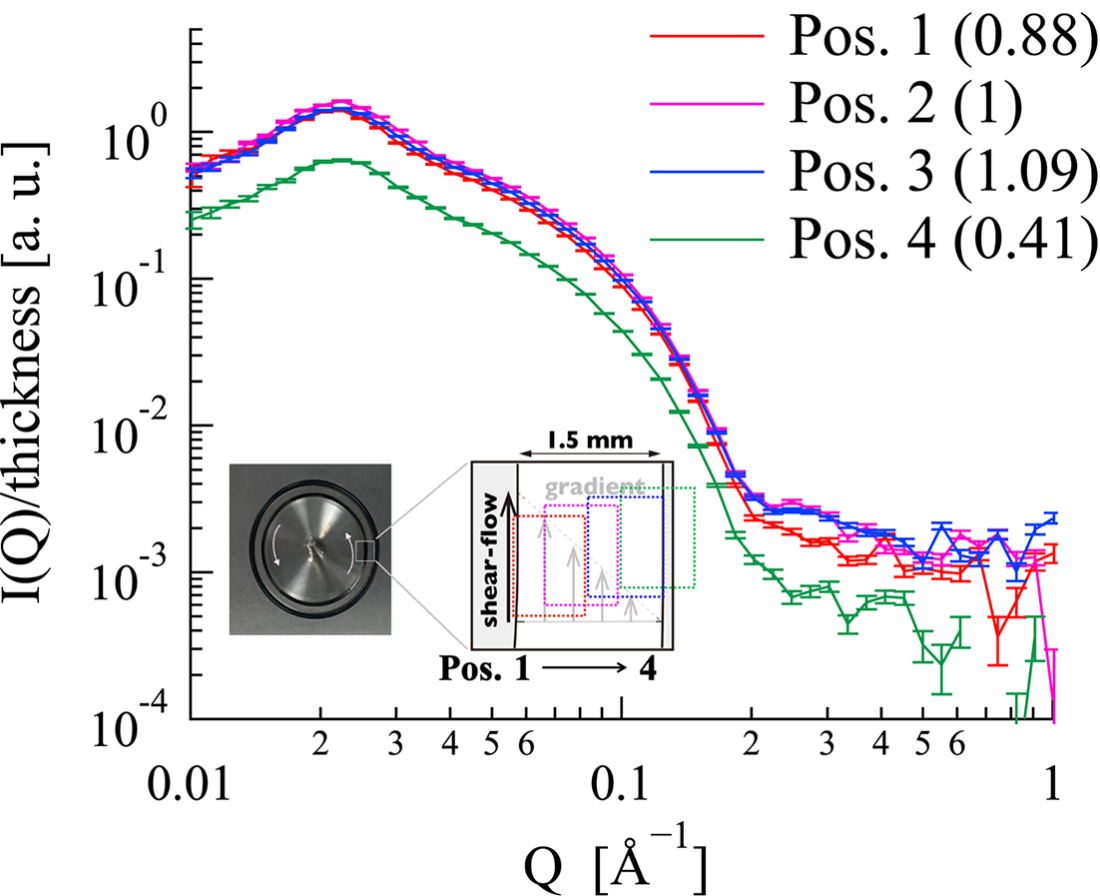
Position dependence of circular-averaged SANS profiles for 12–2–12 in an aqueous solution at 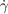 = 0 s^−1^. The SANS profiles were normalized by sample thickness. The inset illustrates the expected gradient of the shear flow with respect to the beam irradiation positions within the 1–2 shear cell gap. The beam irradiation areas are shifted systematically in the vertical direction to avoid overlap. The distance between the centers of each position in the horizontal direction is 0.23 mm.

**Figure 10:**
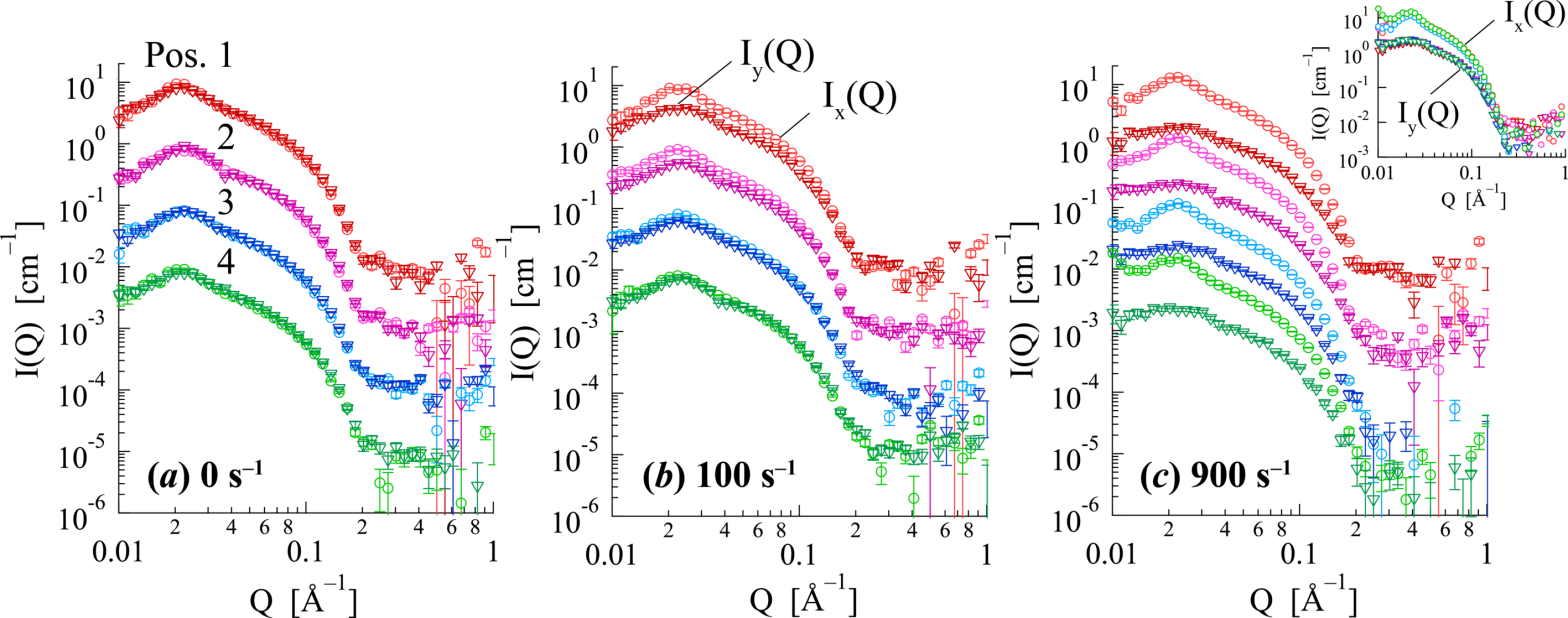
Position dependence of the sector-averaged SANS profiles for 12–2–12 in an aqueous solution at a volume fraction (φ) of 0.013 at (*a*) 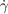 = 0 s^−1^, (*b*) 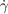 = 100 s^−1^, (*c*) 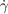 = 900 s^−1^. The 2D SANS profiles (in Fig. 8 ▸) were sector-averaged with the azimuthal angle of ±15°.

Fig. 9 ▸ shows the position dependence of circular-averaged SANS profiles for the 12–2–12 in an aqueous solution at 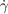 = 0 s^−1^, normalized by sample thickness. While the *Q* dependence of the SANS profiles demonstrated consistency across all measurement positions, significant variations were observed in SANS intensities. Specifically, the SANS intensity at position 4 was very weak compared with those at other measurement positions. When the intensity was normalized to position 2 (as indicated in the figure legend), the resulting scattering intensity values, which reflect the irradiated area, revealed that the SANS profiles at position 4 are equivalent to 41% of the intensity at position 2. This was attributed to the proximity of position 4 to the Cd shielding in front of the 1–2 shear cell. The inset of Fig. 9 ▸ illustrates the expected gradient of the shear flow with respect to the beam irradiation positions within the 1–2 shear cell gap, as determined from the results in Fig. 9 ▸. The sector-averaged SANS profiles of the 12–2–12 solution are shown in Fig. 10 ▸. At 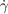 = 0 s^−1^, all SANS profiles were isotropic, and *I*_*x*_(*Q*) and *I*_*y*_(*Q*) were identical, irrespective of the measurement position within the gap. This finding is consistent with a randomly oriented micellar structure under stationary conditions.

In contrast, at 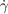 = 100 s^−1^ [Figs. 8 ▸(*b*) and 10 ▸(*b*)], the SANS profiles exhibited a clear position dependence. The SANS intensity showed strong anisotropy near the rotor and gradually became isotropic towards the periphery of the flow channel. This behavior is attributed to the velocity gradient across the gap, where the local shear rate is highest near the moving rotor (position 1) and decreases towards the stationary wall (position 4). The observed WLM alignment varies according to this local shear rate gradient. This spatially resolved observation of varying structural anisotropy within the shear-thickening regime is a key finding enabled by the 1–2 shear plane measurement geometry. At 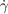 = 900 s^−1^ [Fig. 8 ▸(*c*)], well into the shear-thinning region, the SANS profiles at all positions exhibited strong anisotropy, indicating a highly oriented state of WLMs throughout the gap. As shown in the inset of Fig. 10 ▸(*c*), the sector-averaged SANS profiles were nearly identical across all positions, suggesting no position dependence in the 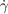 range where shear thinning was observed.

**Figure 11:**
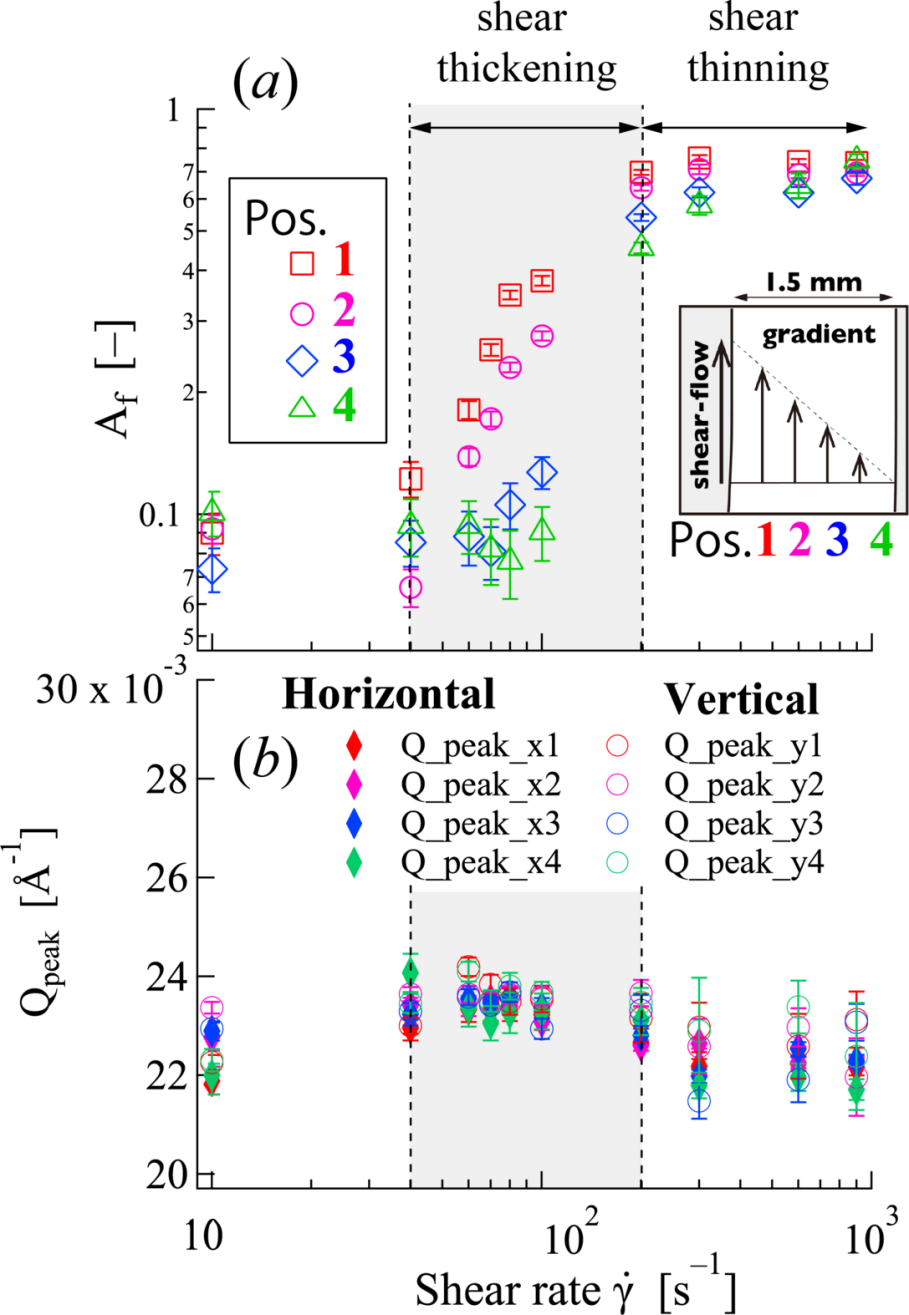
(*a*) Shear rate dependence of the alignment factor 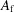 and (*b*) peak position (*Q*_peak_) for 12–2–12 in an aqueous solution at four positions. The 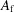 values were obtained by equation (2).

Fig. 11 ▸(*a*) shows the shear rate dependence of the *A*_f_ value, evaluated using equation (2) at each of the four measurement positions. The same behavior as observed in position 2 was seen across all positions. Consistent with the 2D patterns (Fig. 8 ▸), at low shear rates (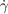 < 40 s^−1^), the *A*_f_ values were low and similar for all positions, reflecting the random orientation of WLMs throughout the gap. As the shear rate increased into the shear-thickening regime (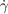 ≃ 40–200 s^−1^), *A*_f_ increased significantly, indicative of developing micellar alignment. Crucially, within this regime, *A*_f_ also exhibited a strong dependence on position: higher *A*_f_ values were observed closer to the moving rotor (position 1), and these values progressively decreased towards the stationary wall (position 4). This observation clearly demonstrates that the degree of shear-induced micellar orientation is non-uniform across the gap during shear thickening. In contrast, during the shear-thinning regime (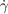 > 200 s^−1^), *A*_f_ values were high and nearly constant across all positions, indicating a uniformly highly oriented state of micelles throughout the gap.

The position dependence of the characteristic intermicellar distance was further investigated by evaluating the peak position (*Q*_peak_) from the *I*_*x*_(*Q*) and *I*_*y*_(*Q*) profiles at each of the four positions across the gap as a function of shear rate [Fig. 11 ▸(*b*)]. As can be observed, the *Q*_peak_ values remain remarkably constant across the four measurement positions. Furthermore, the *Q*_peak_ values themselves remain largely constant with increasing shear rate, with only a slight tendency to decrease (indicating a slight increase in intermicellar spacing) in the high shear rate shear-thinning regime. The lack of notable position dependence of *Q*_peak_, even in the shear-thickening regime where *A*_f_ exhibits substantial spatial variation [Fig. 11 ▸(*a*)], indicates that the average intermicellar spacing is relatively consistent across the gap. This indicates that local changes in micelle concentration are not the predominant cause of position-dependent alignment.

#### 3.2.2. Transmission

Macroscopic concentration fluctuations, such as shear bands, are often associated with complex rheological behaviors in surfactant solutions (Liu & Pine, 1996 ▸; Decruppe *et al.*, 2001 ▸; Salmon *et al.*, 2003 ▸; Miller & Rothstein, 2007 ▸; Sambasivam *et al.*, 2015 ▸). To investigate the possibility of macroscopic concentration fluctuations in the 12–2–12 solution, neutron transmission through the sample was measured. The transmission of the sample solution is predominantly determined by the number of hydrogen atoms, as hydrogen has the largest scattering cross section among all atoms in the 12–2–12 solution. It is known that the H_2_O ratio in D_2_O/H_2_O mixtures can be evaluated from transmission values (May *et al.*, 1982 ▸). At TAIKAN, the transmission is detected simultaneously with the scattering.

**Figure 12:**
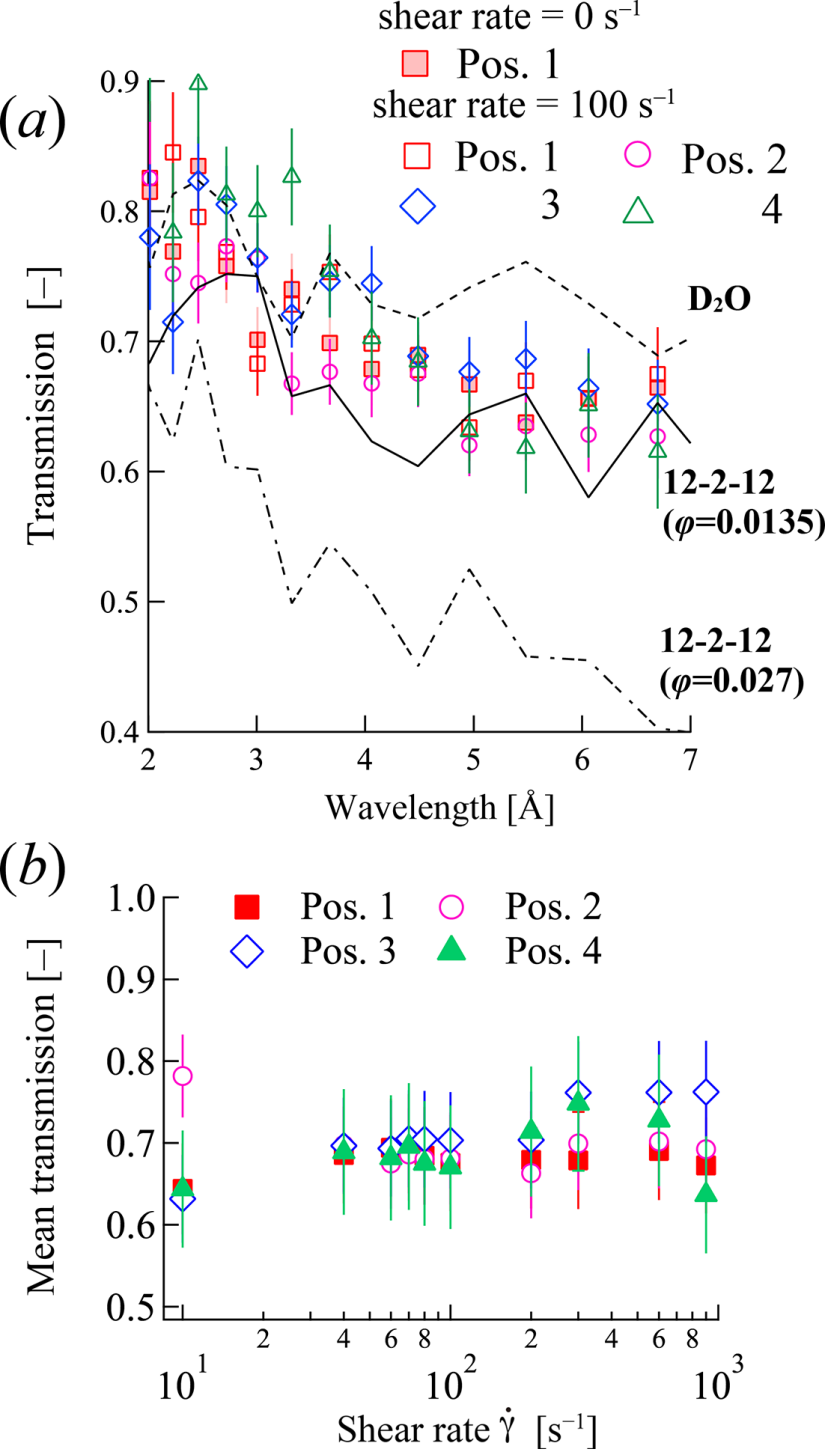
Wavelength dependence of neutron transmission for 12–2–12 in an aqueous solution at four positions. The transmission and SANS were detected simultaneously during the measurement.

Fig. 12 ▸(*a*) shows the wavelength dependence of the transmission measured simultaneously with the SANS at a shear rate of 100 s^−1^. Consequently, the transmission value was directly related to the surfactant volume fraction. The wavelength dependence of the transmission of the 12–2–12 solutions at φ = 0, 0.0135 and 0.027 is shown in Fig. 12 ▸. The transmission gradually decreased as the sample volume fraction increased. A clear position dependence was observed in the SANS profile for the 12–2–12 solutions, whereas the transmission remained nearly uniform. To further investigate this with improved statistical precision, the mean transmission was calculated over the wavelength range of 3 to 6 Å. Fig. 12 ▸(*b*) shows the shear rate dependence of the mean value of transmission of the 12–2–12 solutions at each of the four positions across the gap. These mean transmission values are largely uniform across all four positions for both the quiescent state and under shear at 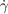 = 100 s^−1^, within experimental uncertainty. No significant or systematic variation in transmission with position was detected that would suggest the formation of distinct shear bands with notably different surfactant concentrations.

The transmission results were consistent with the largely uniform *Q*_peak_ values across the gap [Fig. 11 ▸(*b*)], suggesting that the shear thickening observed around 100 s^−1^ is primarily associated with the shear-induced changes in wormlike micellar structure (elongation and orientation) and the spatial variation of the WLMs, as revealed by SANS, rather than being dominated by macroscopic concentration fluctuations. However, it is important to recognize that, at the relatively low surfactant concentration used in this study (φ = 0.0135), the sensitivity of transmission measurements to subtle concentration changes is limited. While our data do not support strong macroscopic shear banding, the possibility of more subtle concentration variations cannot be entirely excluded on the basis of the transmission data alone. Nonetheless, the consistency of *Q*_peak_ across the gap further supports the idea that large variations in local micelle density are unlikely.

Therefore, considering the SANS results together with the transmission data, the distinct position dependence of WLM orientation [Fig. 11 ▸(*a*)], in the absence of clear macroscopic concentration fluctuations, suggests that the structural response of the micelles to the local shear rate within the velocity gradient is the primary factor driving the observed spatial inhomogeneity during shear thickening. The micellar structuring process, likely involving elongation and subsequent orientation, appears to be highly sensitive to these local shear conditions, leading to the observed non-uniform alignment across the gap.

## 4. Conclusions

In this study, we investigated the shear-induced structural evolution of WLMs formed by the gemini-type cationic surfactant 12–2–12 in an aqueous solution, focusing specifically on the velocity–velocity gradient (1–2) shear plane. By performing SANS measurements at four different positions across the flow channel of a 1–2 shear cell, we successfully characterized the spatial dependence of WLM structures under varying shear rates, encompassing both shear-thickening and shear-thinning regimes.

The key findings demonstrate that during the shear-thickening process (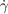 ≃ 70–200 s^−1^) a significant position-dependent alignment of WLMs occurs across the gap, with higher orientation near the moving rotor, indicating a strong sensitivity of micellar structural response to the local shear rate. Despite this pronounced spatial variation in alignment, the intermicellar distance, inferred from the SANS peak position (*Q*_peak_), remained largely uniform across the gap, even under conditions of strong orientational gradients (*e.g.* at 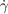 ≃ 100 s^−1^). Furthermore, simultaneous neutron transmission measurements, corroborated by the consistent *Q*_peak_ values, did not reveal significant macroscopic concentration fluctuations within the shear-thickening regime. These collective results strongly suggest that the observed shear thickening is primarily driven by shear-induced micellar elongation and orientation, and the spatial inhomogeneity of the WLMs, rather than by distinct shear banding involving significant concentration differences.

These observations in the 1–2 plane are broadly consistent with and complementary to our previous Rheo-SANS investigations of the same 12–2–12 system in the 1–3 and 2–3 shear planes (Iwase *et al.*, 2019 ▸). However, this new study uniquely provides direct experimental evidence of the spatial inhomogeneity of micellar alignment within the velocity gradient, offering crucial insights into how local shear conditions govern the structural transitions underlying macroscopic rheological behavior. The capacity to address these position-dependent structural intricacies underscores the efficacy of SANS measurements in the 1–2 shear plane, facilitating a more comprehensive, 3D understanding of complex fluids under shear. This approach has significant potential for elucidating the intricate relationships between nanostructure, local flow kinematics and macroscopic rheology in a wide range of soft matter systems. Future investigations could extend this methodology to other shear-thickening systems or explore shear-thinning phenomena in more concentrated micellar solutions, further leveraging the capabilities of SANS measurements in the 1–2 shear plane to unravel complex flow behaviors.
